# Book Reviews

**Published:** 1903-01

**Authors:** 


### BOOK REVIEWS.

A TEXTBOOK OF THE SURGICAL PRINCIPLES AND SURGICAL DISEASES OF THE FACE, MOUTH AND JAWS. For Dental Students. By HORACE GRANT, A. M., M. D., Professor of Surgery and of Clinical Surgery, Hospital College of Medicine; Professor of Oral Surgery, Louisville College of Dentistry, Louisville. Octavo volume of 231 pages, with 68 illustrations. Philadelphia and London: W. B. Saunders & Co. 1902. [Cloth, $2.50 net.]

The great need of improvement in the methods of teaching dental students, a plea for which has been made many times, receives additional emphasis in this excellent textbook. The practice of dentistry is becoming a scientific occupation and the teaching necessary to prepare a student for its arduous work must be thorough-going and complete. The contribution of this work to the literature of scientific dentistry will serve to advance the standard in the direction named.

Professor Grant begins his book very properly with a chapter on bacteriology, which it would be well for every student of dentistry to study with care. There is too much laxity in the matter of sterilisation in dental offices, and this chapter without doubt will stimulate greater care in this direction. Another chapter for dentists to observe with attention and to heed is the one devoted to the consideration of anesthesia. The accidents from carelessness in the administration of anesthetics in dental offices are too numerous, and the timely presentation of the subject here is to be commended. Another chapter of importance is the one on syphilis. This topic should be studied with great care by dentists for here, again, is an opportunity for improvement in preventive measures. These three chapters are worth the price of the book and the information they contain ought to be widely disseminated among dentists.

The chapter on hemorrhage is of great interest, not only because it deals with an important subject but because, further, it handles it in an able manner. Besides the topics alluded to there are others of importance and great interest that are well presented; but these mentioned being of fundamental significance have been pointed out, to afford the intending purchaser an idea of the scope of the work. There are twenty-nine chapters in all which discuss inflammation, suppuration, ulceration, gangrene, autoinfection, pyĕmia, wounds, tumors, tuberculous and other diseases of bones; surgical lesions of the mouth and face, cleft palate, harelip, lesions of the lips and tongue, diseases of the salivary glands, of the maxillary and other sinuses, neuralgia, dislocations and fractures of the jaw and bones of the face, together with other topics, or subtopics of interest and value.

The illustrations, while not numerous, serve to delineate the text here and there with clearness, though there is room for improvement in this direction both in quality and quantity. A

well-arranged index serves to round out a most excellent treatise upon which the distinguished author deserves the congratulations of both dental and medical professions.

A TREATISE ON THE PRINCIPLES AND PRACTICE OF GYNECOLOGY. By E. C. DUDLEY, A. M., M. D., Professor of Gynecology in the Northwestern University Medical School, Chicago. New (3d) edition enlarged and thoroughly revised. Octavo of 756 pages, with 474 engravings, of which 60 are in colors and 22 colored plates. Philadelphia and New York: Lea Brothers & Co. 1902. [Cloth, $5.00 net; leather, $6.00 net; half morocco, $6.50 net ]

The favor Dudley's treatise met with when first issued is indicated by the exhaustion of two editions in but little more than three years. Considering that many works on gynecology have been issued within a comparatively recent date, this is strong testimony as to the value of Dudley's book.

In the journal for December, 1898, p. 314, we pointed out some of the essential features of this treatise—its stronger points—and gave reasons for its superior worth. The general scope and plan of the volume, too, were considered in much detail. Now, after three and a half years of trial and having stood the test, there is small need of a repetition of the method adopted in our first review. It is more particularly our duty at this time to indicate salients, changes, and improvements.

In the first place it will be remembered that Dudley's aim is to present a practical treatise divided into sections or chapters from the viewpoint of pathological and etiological sequence, not on the basis of the diverse diseases of special organs without, however, sacrificing the unity of the pathological processes as they may affect consecutively the different pelvic organs. Besides the plan of grouping subjects on pathological lines, Dudley also seeks to exclude whatever is not based upon practical experience; in other words, he sets forth that which is of greatest aid to the student and physician. He has dealt with the whole field, as will thus become apparent, from the standpoint of originality, rather than tradition, and the work thus becomes of the greatest possible value.

The book has been thoroughly revised in a most painstaking manner, obsolete material being excluded and new matter added, many chapters being in this way arranged, rewritten, or condensed, as the case may be. This has served to add about 100 pages without making the volume cumbersome. Tables and parallel columns are extensively used, which serves to minimise space while enlarging the usefulness of the book for class work and reference.

The illustrations deserve commendation as being clear, original, and helpful to a better appreciation of the text. Dudley in this respect demonstrates, again, his thoroughly practical method of teaching, being a master of his subject, and possessing a peculiarly gifted talent for demonstrating it to others.

---

Diseases of the Rectum and Anus. Designed for Students and Practitioners of Medicine. By Samuel Goodwin Gant, M. D., L.L. D., Professor of Rectal and Anal Surgery at the New York Post-Graduate Medical School and Hospital; Attending Surgeon for Rectal and Anal Diseases to the New York Post-Graduate Hospital, St. Mark's Hospital, Hebrew Sheltering Guardian Orphan Asylum, and New York Infant Asylum. Second edition, rewritten and enlarged, with 37 full-page plates, 20 of which are in colors, and 212 smaller engravings and half-tones. Pages xxiv.-687. Royal octavo. Philadelphia: F. A. Davis Company. 1902. [Extra cloth, $5.00 net; sheep or half-Russia, $6.00 net, delivered.]

According to the title page, of which the foregoing is a transcript, we are told this is a second edition of a work previously written by this author, but a comparison of the two editions discovers a surprising difference between them; indeed, it is so great as to almost justify the conclusion that they are altogether different books, with a mere coincidence of authorship. The first is a somewhat crude example of provincialism in book writing, while the second is a splendid specimen of metropolitan development.

It is true that the six years that have passed between the issuance of the two editions have witnessed many improvements in this department of medical science, but it is also quite true that the author, having removed from the West to New York, has expanded his methods and contracted his statements, so that now his book takes place among the foremost of its kind. It is an exposition of the present state of knowledge concerning diseases of the region with which it deals, and is a credit to the author and to American medicine.

In this edition, besides the rewriting of the old book, the author has added new chapters and new engravings, until the original plan of the work is lost in the new and splendid book that has been evolved. It now takes a high rank among the textbooks of the period, and is a safe guide—to undergraduates as well as practitioner—to the study of diseases of the lower intestinal tract and region, as well as to their cure by operative surgery or other means.

The illustrations deserve to be mentioned as a special feature of the book. They are of the best, and represent the very foremost point of advance in the illustrator's art. The author deserves the congratulations of his professional colleagues for the improvements he has made in this edition, and for the general excellence of his treatise.

---

A TEXTBOOK OF ANATOMY. By American authors. Edited by FREDERIC HENRY GERRISH, M D., Professor of Anatomy in the Medical School of Maine, Bowdoin College. Second Edition, thoroughly revised and enlarged. In one imperial octavo volume of 943 pages, with 1003 engravings in black and colors. Philadelphia and New York: Lea Brothers and Co., Publishers. 1902. [Cloth, $6.50 net; leather, $7.50 net; flexible waterproof binding, for use on the dissecting table, $7.00 net.]

A good anatomical textbook is a necessary part of every physican's library and of every student's outfit. Moreover, the most practical book is made up into a single volume which deals with the essentials of the human body, leaving out exceptional conditions and unimportant departures from the normal state. Gerrish's anatomy is just such a work.

Let us hear the editor speak on the subject, which he does in the following words:

> Between the extremes, represented on the one hand by pocket manuals, with their flavorless condensation, and on the other hand, by encyclopedias of universal inclusiveness, there is room for a work of convenient size, sufficient to contain in systematic array those portions of anatomical knowledge which are necessary to the intelligent study of physiology, surgery, and internal medicine. * * * The authors have unceasingly endeavored to facilitate the work of both student and teacher. Both are tested in every examination, and particularly in those conducted by state licensing boards. Accordingly, from the vast accumulations of anatomical science, those portions have been selected which are likely to be of actual service to the student in his subsequent study, and to the practitioner in his clinical work.

This extract from the preface expresses in terse English the scope and purpose of the work, and is in fact a compact review in and of itself. In the preparation of the work the editor has associated with himself some of the ablest anatomists in America. The work of all the contributors is so blended that the effect is homogeneous and quite like that of a single author. Were it not for the announcement of the names at the head of the chapters in the table of contents it would be impossible to differentiate their work. While, therefore, the book is encyclopedic in fact, it is monogenetic in effect.

The illustrations are marvels of richness in detail and color, being over 1,000 in number, executed by the most skilful artists. They lend attractiveness to the text and enhance the instructive character of the work in a manifold degree. It is an anatomical exposition that is not excelled in practical value, and appeals to student, teacher and practitioner alike, in its wealth of material, accuracy and artistic merit of illustration, and in the beauty of mechanical make-up of the volume.

---

THE INTERNATIONAL TEXTBOOK OF SURGERY. In two volumes. By American and British Authors. Edited by J. COLLINS WARREN, M. D., LL. D., F. R. C. S., (Hon.), Professor of Surgery, Harvard Medical School; and A. Pearce Gould, M. S., F. R. C. S , of London, England. Second edition, thoroughly revised and enlarged. Vol. I. General and Operative Surgery. Royal octavo of 965 pages, with 461 illustrations and 9 full-paged colored lithographic plates. Vol. II., Special or Regional Surgery. Royal octavo of 1,122 pages, with 499 illustrations, and 8 full-paged colored lithographic plates. Philadelphia and London: W. B Saunders & Co. 1902. [Cloth, $5.00 net; sheep or half-morocco, $6.00 a volume net.]

It is not surprising that so good a treatise as this should pass through its second edition in but little more than two years. When first issued it was recognised as entitled to a foremost place as a textbook, and it bids fair to retain that position for some time to come.

The entire work has been subjected to careful revision though so completely had the original work been done that few changes have been required.

Military and naval surgery have received a new impetus from the Spanish-American and South African wars, and this fact has been taken advantage of by the authors in the revision and rewriting of the chapters on these subjects.

The chapters on diseases of the lymphatic system, on the surgery of the spleen, and on the surgery of the kidney, have undergone revision, so that the recent surgical improvements relating to these organs may be brought forward, recorded, and given place in the literature of the present day.

The general scope and plan of the work remains the same. In the first volume is given the essentials of general and operative surgery, and in the second regional surgery is detailed. The engravings have been increased in number, making one of the most copiously illustrated textbooks extant. The American author is one of the most accomplished and best known surgeons of the period, and his English colleague is scarcely less distinguished. It is a fortunate editorial combination, because it serves to present the best surgery of the hour on both sides of the Atlantic to English-speaking people in every quarter of the globe.

The Treatment of Fractures. By Charles L. Scudder, M.D., Assistant in Clinical and Operative Surgery, Harvard Medical School. Third edition, revised and enlarged. Octavo, 480 pages, with 645 original illustrations. Philadelphia and London: W. B. Saunders & Co., 1902. [Polished buckram, $4.50 net; half morocco, $5.50 net.]

The popularity of this monograph, great as it became at once upon the appearance of the first edition, has gone on increasing with such amazing rapidity, that it is seen in its third edition in but little more than two years. Nor is it strange, for it is easily the most attractive treatise that has been published on the treatment of fractures and, moreover, it may be called, without exaggeration or reflection on other good works, the most instructive. This is partly because of its illustrations, and partly because of the direct and uncomplicated methods in presenting each fracture in detail.

The illustrations are something phenomenal. In a work of 485 pages there are 645 pictures, in half-tone and wood, most of which are from life-models, showing dressings in various stages of application, and in numerous instances the fracture itself is depicted before reduction, thus presenting its deformities and peculiarities; so that, altogether it is the best illustrated medical book, whether judged from quantity or quality, that has been published within our knowledge.

The author has made some changes and additions in this edition, among which are facts in gunshot fractures developed by the recent wars, some of the plates being taken from Mr. Makins's "Surgical Experience in South Africa." There is every reason to believe that the distinguished young author will be called upon at an early date to prepare another edition of this most valuable treatise, for the probabilities are very great that this one will be exhausted within a comparatively short time.

---

International Clinics. A Quarterly of Illustrated Clinical Lectures and especially prepared Articles on Medicine, Neurology, Surgery, Therapeutics, Obstetrics, Pediatrics, Pathology, Dermatology, Diseases of the Eye, Ear, Nose, and Throat, and other Topics of Interest to Students and Practitioners by leading Members of the Medical Profession throughout the World. Edited by Henry W. Cattell, A. M., M. D., Philadelphia, with the Collaboration of John B. Murphy, M. D., Chicago; Alexander D. Blackader, M. D., Montreal; H. C. Wood, M. D., Philadelphia; T. M. Rotch, M. D., Boston; E. Landolt, M. D., Paris; Thomas G Morton, M. D., Philadelphia; James J. Walsh, M. D., New York; J. W. Ballantyne, M. D., Edinburgh, and John Harold, M. D., London, with Regular Correspondents in Montreal, London, Paris, Leipsic, and Vienna. J. B. Lippincott Company, Philadelphia and London. [Cloth, $2.00.] Volume III. [Twelfth Series, 1902.]

This volume discusses topics in the fields of therapeutics, medicine, surgery, obstetrics and gynecology, and diseases of the eye, ear and throat. Two special articles are added, one on the function of the digestive glands based on the researches of Paolof and his pupils, by Peter Borrissof; and the other being a critical study of the theory of inflammation, by Hans Schmaus.

Among the other articles of note in the book may be mentioned one on the treatment of morphinism, by Crothers; another on the insect pests of human beings, by Walsh; and yet another on the effects of firearms at short range, by Brinton. Moreover, the book is filled with lectures of interest and contains many illustrations of merit. It is an excellent number in the series, and the clinics deserve the continued support of the profession.

---

A TEXTBOOK OF PHYSICAL DIAGNOSIS. For Students and Practitioners of Medicine. By EGBERT LE FEVRE, M. D., Professor of Clinical Medicine, and Associate Professor of Therapeutics, University and Bellevue Medical College, Attending Physician to Bellevue and St. Luke's Hospitals, New York, 12mo, 440 pages, with 74 engravings and 12 plates. Lea Brothers & Co.. Philadelphia and New York. 1902. [Cloth, $2.25 net.]

Among the additions that have been made recently to an already extensive literature relating to physical diagnosis this book will take front rank. It is prepared by a teacher of experience and a clinician of accomplishment. In this book Le Fevre gives adequate instruction upon all the details of physical diagnosis—inspection, palpation, percussion, and auscultation—together with all other directions for obtaining information in diseases of the respiratory and circulatory systems. The abdomen, too, receives equally detailed attention and the same methods as applied to the thorax are employed and explained, with the variations necessary to be made in the diagnosis of diseases of organs of the lower cavity. A number of engravings and *x*-ray plates are presented to elucidate the text, and an excellent index closes a valuable book that is a distinct addition to the literature useful to the student and junior practitioner of medicine.

BLAKISTON'S QUIZ COMPENDS. Compend of Special Pathology. By ALFRED EDWARD THAYER, M. D., Assistant Instructor in Cross Pathology, Cornell Medical College; Pathologist to the City Hospital, New York. Duodecimo, pp. 322. Illustrated. Philadelphia. P. Blakiston's Son & Co. 1902. [Price, 80 cents net.]

The importance of a knowledge of pathology increases with the advancement of medical science along general lines. Any method or book, or picture that tends to improve or increase this knowledge is to be welcomed.

Experience would seem to indicate that these little volumes are of value in this direction. They are prepared by teachers and are purchased by students upon their advice, hence must be accepted as part of the necessary equipment of the undergraduate. This book is one of the very best of the compends and is prepared by a teacher who not only understands his subject but imparts information concerning it with surprising clearness. For these reasons it is to be commended to the beginner.

